# Review on synthesis, properties and multifarious therapeutic applications of nanostructured zirconia in dentistry

**DOI:** 10.1039/d2ra00006g

**Published:** 2022-04-27

**Authors:** Ranjeet A. Bapat, Ho Jan Yang, Tanay V. Chaubal, Suyog Dharmadhikari, Anshad Mohamed Abdulla, Suraj Arora, Swati Rawal, Prashant Kesharwani

**Affiliations:** Faculty, Division of Restorative Dentistry, School of Dentistry, International Medical University Kuala Lumpur 57000 Malaysia; Postgraduate Student, Department of Restorative Dentistry, University of Malaya 50603 Kuala Lumpur Malaysia; Faculty, School of Dentistry, DY Patil Deemed to be University Navi-mumbai-400706 India; Faculty, Department of Pediatric Dentistry and Orthodontic Sciences, King Khalid University Abha Kingdom of Saudi Arabia; Faculty, Department of Restorative Dental Sciences, King Khalid University Abha Kingdom of Saudi Arabia; Faculty, Director, Predoctoral Periodontology, Marquette University Milwaukee WI 53201-1881 USA; Department of Pharmaceutics, School of Pharmaceutical Education and Research Jamia Hamdard New Delhi-110062 India prashantdops@gmail.com https://scholar.google.com/citations?user=DJkvOAQAAAAJ&hl=en +91-7999710141 +91-7999710141

## Abstract

Amongst dental ceramics, nano zirconia (ZrNp) has shown exceptional developments in the field of dentistry in recent years. Zirconia is an oxide that possess superior optical, mechanical, and biological properties. As a novel nanoparticle, it has been widely used in various fields of dentistry due to its improved mechanical properties, biocompatibility, and stable structure. Provision of metal free solutions is one of the prime requirements in dental materials. Many metal alloys used extensively possess unaesthetic colors and display chemical interactions in the oral cavity encouraging use of zirconia for dental use. Use of ZrNp based ceramics has increased due to its resistance to corrosion, superior color matching that enhances esthetics and improved strength compared to conventional biomaterials. This review discusses the recent scientific literature on the synthesis, properties and types, applications, and toxicity of ZrNp in the field of dentistry.

## Abstract 2

Amongst dental ceramics, nano zirconia (ZrNp) has shown exceptional developments in the field of dentistry in recent years.

## Graphical abstract

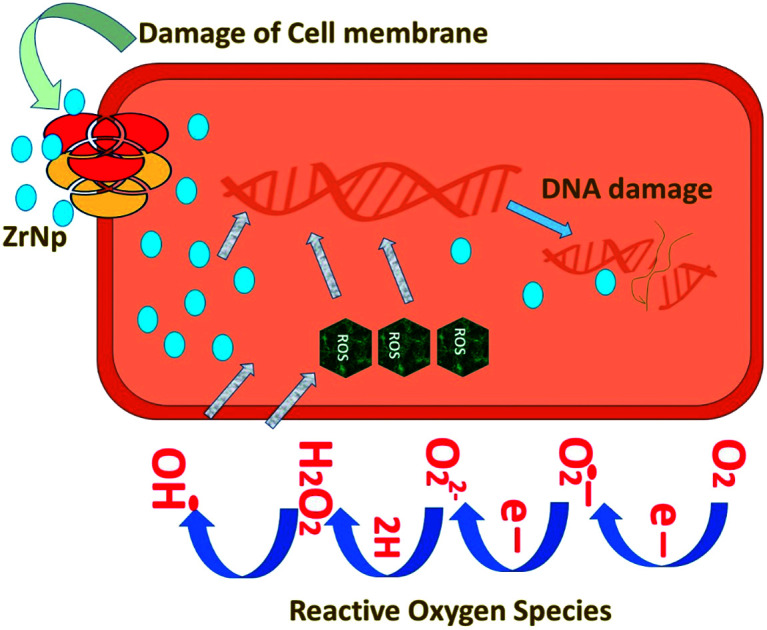

## 1. Introduction

Since the 1960s zirconia has been used as a biomaterial for biomedical applications. Zirconium dioxide (ZrO_2_) occasionally called zirconia is a white crystalline oxide of zirconium. The crystalline content of ZrO_2_ is approximately 96–99% with no glassy phase, resulting in increased flexural strength, fracture toughness, and hardness, increased mechanical properties, satisfactory esthetics, and excellent biocompatibility, consequently expanding its application in dentistry. Its natural white color, stable chemical properties, superior resistance to corrosion, and biocompatibility with implant materials, make it an effective high-performance ceramic material. Due to its biocompatibility, high fracture toughness and radiopacity, zirconia has been used as a structural material for construction of crowns, bridges, inserts, and implants.^1^ Recently small blocks of ZrO_2_ such as nano powders or micro powders have been used widely in dentistry. ZrO_2_ provides an extended spectrum of application scenarios, as a nano powder filling, nano coating, and sintering raw material. By employing ZrNp, bionics and mechanical properties of dental ceramics and tissue engineering scaffolds can be greatly enhanced. Recently concluded studies indicated that the incorporation of ZrNp can significantly increase the mechanical characteristics of the materials. The biocompatibility of solid surfaces is increased by coating them with ZrNp, thereby providing a nanostructured surface.

ZrNp is widely used in dentistry due to its toughness and superior strength. Zirconia's high chemical stability, biocompatibility, suitable fracture resistance and flexural strength has led to its attraction as a dental implant material or coating. Application of ZrNp nano-technology treatment includes acid etching, plasma spraying and bioactive layer deposition which elevates the surface hydrophilic characteristics of dental implants.

Bone tissue engineering is a combination of biomaterial scaffolds, cells, engineering methods, and physical and chemical factors for restoring, maintaining, or improving function. Good mechanical strength of the biomaterial scaffold is necessary to meet the requirement of the stressed zone. For creating an environment which supports the growth of osteoblasts, vessels, and new bone, these scaffolds are required to have good cytocompatibility. For satisfying cytocompatibility and high strength, ZrNp could be a good fit as a filling material in composite scaffold.^2^ Overall, ZrNp can be employed in dental ceramics, implants, denture basement and tissue engineering.

## 2. Synthesis, types, and properties of ZrNp

### 2.1. Synthesis

Nano zirconium oxide, a metal ceramic oxide provides varied advantages as a dental biomaterial. Admirable natural white color, enhanced toughness, exceptional strength, superior high-temperature thermal stability and fine corrosion resistance are few of its qualities which makes it an infallible restorative and prosthetic material for today's dentists. ZrO_2_ has 3 different crystal structures: cubic (c-ZrO_2_), tetragonal (t-ZrO_2_) and monoclinic (m-ZrO_2_), and all of these structures are stable at different range of temperatures. Wet-chemical synthesis approaches like coprecipitation^2–4^ hydrothermal synthesis,^5^ sol–gel preparation^6^ are used for the synthesis of ZrNp powders nowadays. Wet-chemical synthesis holds a significant advantage over the physical method as it can fulfill the specification of nano size, which the physical method fails to do so. Gas-chemical method is another method which is not used often because of its high costs of production.^2^

#### 2.1.1. Coprecipitation method

It is a standard method for the synthesis of nanocrystals. This method enables to attain particles with different diameters. This method involves the incorporation of precipitating agent with the mixture solution of water-soluble zirconium salts along with yttrium (stabilizer).^7^ The insoluble hydroxide precipitate obtained following this reaction is dried to get ZrNp powders.

The likelihood to acquire the product in amounts consisting of grams is an inordinate advantage of this method.^8^ Also, this method enables the usage of cheaper and easier accessible equipment's and nano-particle precursors.^7,9^ However, this method also has its disadvantages. The inefficacy to regulate the size of the nanoparticle product is one of them. To tackle the issue of agglomeration of nanoparticles, Wang *et al.* effectively used ethanol instead of water for the manufacture of ZrNp formulated on direct precipitation.^9^ Coprecipitation technique is uncomplicated, but the purity of the final product is dependent on multiple factors like washing solvent, pH value and drying method; which could adversely affect the sintering characteristics of the nanopowders.^2^

#### 2.1.2. Sol–gel method

It is a type of chemical synthesis which uses comparatively lower temperature to synthesize solid materials from small molecules. The precursors undergo specific chemical reactions like hydrolysis and condensation and get converted into the colloidal or polymeric solution. The dehydrated colloidal solution of material hydroxide particles forms the basis of conversion of sol into gel, a gelatinous substance. Starting materials used to carry out these reactions are mostly metal alkoxides or metal chlorides.^10^ These initial precursors could affect the drying or firing behavior of sol–gel. Successful synthesis of ZrNp was done by Shukla and Seal^11^ using this method. This method requires a lesser processing temperature in comparison with gas chemical methods, is simple to use and has low equipment cost. Furthermore, the microstructure of nano particles synthesized using sol–gel method has a high degree of homogeneity, hardly produces any toxic waste and can receive color layers. However, the drawbacks of sol–gel method are the use of costly chemical reagents and the imperative stability of technological parameters.

#### 2.1.3. Hydrothermal method

Hydrothermal method enables the synthesis of inorganic materials especially particles varying from nano-size to submicron crystals. Here starting materials are subjected to increased temperature and pressure where it undergoes a chemical reaction. The solution is aqueous. In the hydrothermal method, the transformation agent for pressure, heat and mechanical energy are the vapors or fluids.^12^ These attributes enables the production of nanoparticles having uniformity in microstructure, shape and components; thereby, making this a method of choice to manufacture fine particles having controlled forms.^2^ High yield hydrothermal precipitation procedure was used by a group of researchers for the synthesis of zirconia polycrystals and the size of the crystals ranged around 8–10 nm.^13^ The fact that nanoparticles of a small dispersion particle size, top quality, and increased purity can be produced by carrying out this process could at a low temperature is a serious advantage of this method. Additionally, this method also provides the capacity to regulate the shape of grains and nucleating agents.^14^ However, the hydrothermal method requires an apparatus with a high level of complexity and thereby involves high cost.

Between physical, wet-chemical and gas-chemical methods, wet chemical synthesis of ZrNp seems to be the most balanced option in terms of quality control and production costs, but some shortcomings associated with the individual methods (co-precipitation, sol–gel and hydrothermal) demand that users be familiar with the manufacturing techniques as well as the correct indications.

### 2.2. Types

Zirconium oxide crystals has 3 crystallographic phases (Table 1), (a) cubic phase: cubic phase has the shape of a straight prism with square sides. It is stable above 2370 °C and has decent mechanical properties.^15^ (b) Tetragonal phase: tetragonal phase also has the shape of a straight prism but with rectangular sides. It has improved mechanical properties and is stable between 1170 °C and 2370 °C.^15^ (c) Monoclinic phase: monoclinic phase has the shape of a deformed prism with parallelepiped size. It has reduced mechanical properties and might lead to a decrease in cohesion and density of ceramic particles. It is stable at room temperatures up to 11700 °C.^15^

**Table 1:** Crystallographic phases of zirconium oxide crystals

Crystallographic phases of zirconium oxide crystals	Shape	Stable temperature	Mechanical properties
Cubic	Straight prism with square sides	Above 2370 °C	Decent mechanical properties
Tetragonal	Straight prism with rectangular sides	Range of 1170 °C and 2370 °C	Improved mechanical properties
Monoclinic	Deformed prism with parallelepiped size	At room temperatures upto 11 700C	Reduced mechanical properties

#### 2.2.1. Stabilized zirconia

Most commonly used Zirconia in dentistry is a modified yttria (Y_2_O_3_), tetragonal zirconia polycrystal (Y-TZP) as it has exceptional mechanical properties and tear resistance.^16,17^ Incorporation of Yttria stabilizes the conversion of crystal structure when fired at high temperature and enhances the strength of zirconia.^16^ Monoclinic crystallographic phase of zirconia on heating, starts changing to another crystallographic phase called tetragonal phase and finishes this transformation at 1206 °C, and on cooling this tetragonal phase starts transformation to the monoclinic phase and ends this transformation around 1020 °C, showing a type of phase transformation called as martensitic transformation.^16^ Stabilizing oxides like yttria (Y_2_O_3_) are added to prevent the formation of ceramic cracks that could be formed during this zirconia phase transformation as there is a comparatively large volume change in the unit cell of monoclinic configuration; where it takes up additional volume when compared to tetragonal configuration (around 4%).^16^ The fracture toughness of Y-TZP is 4–5 MPa which is greater than the regular dental ceramics.^17^ This is because the beads in the tetragonal phase are changed from the monoclinic phase which in turn leads to the compression of forces around the defects, halting it's dissemination.^17,18^ Due to its outstanding mechanical properties in comparison to other dental ceramics whose uses were limited to single tooth restorations or small bridges, Y-TZP are used in a wide range of clinical situations like long span bridges in anterior and posterior regions,^19^ a rare aesthetic option that can be used in the lateral area,^17,20^ dental implants^21^ and for post and core restorations.^22^

### 2.3. Properties

#### 2.3.1. Biocompatibility

Numerous *in vitro* and *in vivo* studies have shown that Y-ZTP have excellent biocompatibility without showing any local or systemic adverse reactions.^15^ The reaction of the bone and the resultant inflammation after the zirconia has been placed in the oral cavity is found to be tolerable. *In vitro* tests for zirconia exhibited lesser cytotoxicity than titanium dioxide (TiO_2_) which is comparable with other materials like alumina.^17^

#### 2.3.2. Optical properties

Aesthetics of a dental restoration hinges on its various optical properties like translucency, color, opacity and fluorescence.^17^ Among these properties, an appropriate translucency is of paramount importance to fabricate a naturalistic dental restoration. Translucency is the ability of the material to allow the passage of light through it, during which it absorbs some light and the rest of it is scattered and reflected from its surface.^23,24^ Translucency can be adjusted by regulating the light absorbed, reflected, and transmitted through the material. Voids,porosity and the chemical nature of the crystals contribute to the scattering.^23^ Another factor which contributes to scattering is the crystalline content of the crystals. Crystalline content is usually high, while obtaining higher strength and this generally culminates in the material having a greater opacity.^25^ Consequently, the polycrystalline ceramics exhibiting a high scattering effect in turn shows higher opacity^26^ and lesser translucency when compared with glass ceramics. A study done by Casolco *et al.* showed that restricting the final size of partially stabilized zirconia crystals to 55 nm by limiting the sintered material improves the translucency.^27^

#### 2.3.3. Wear behaviour

Clinical evaluation of the enamel wear caused by ZrNp restorations to the naturally opposing teeth is of vital significance; as the normal wear process (due to factors like salivary pH, bruxism, forces exerted by intraoral musculature, thickness of enamel *etc.*) can get modified while using dental restorations having contrasting wear behavior. Based on the findings of few *in vitro* studies, it was found that polished full zirconia crowns caused least amount of enamel wear on naturally opposing teeth.^28–30^ This is mostly because the polished full zirconia crowns are less abrasive to opposing teeth enamel due to its smooth surface^31^ which in turn makes it more biocompatible when compared with other dental ceramics.^32^ Nevertheless, more *in vivo* studies are needed to validate these findings.

#### 2.3.4. Low temperature degradation (LTD)

Zirconia based ceramics are most popularly used in dentistry due to the plethora of advantages they provide like the aesthetics, biologic compatibility, mechanical properties *etc.* Nonetheless, one disadvantage of it is that it undergoes hydrothermal aging *in vivo*.^33^ This process which is better known as LTD can post significant restrictions to the usage of zirconia based ceramics in dentistry; due to its exposure to saliva and other fluids in the oral cavity along with the stress caused by mechanical forces leading to its failure.^34^

LTD is a phenomenon in which zirconia undergoes an aging process initially at the surface and subsequently spreading to the material depth. The transfiguration of one grain is succeeded by a volumetric enlargement leading to microcracks and alterations in other grains. Proceeding of surface degradation is accentuated by penetration of water and this transfiguration proceeds from one grain to another, leading to microcracking, grain pullout and eventually surface roughening.^17^ These turns of events will invariably compromise the mechanical property of zirconia.

Several studies have illustrated the correlation between the grain size and a resistance to hydrothermal aging. According to these studies the LTD sensitivity can be subdued or reduced by the usage of a nanometric grains' microstructure.^35–37^ A study carried out by Paul *et al.* showed that nanostructured zirconia having a grain size of <100 nm was resistant to LTD sensitivity.^38^ Similar result was achieved by Matsui *et al.* by using a grain size of <200 nm.^39^ These studies show that by reducing the grain size of zirconia-based ceramics to nanoscale can improve its properties.

#### 2.3.5. Mechanical properties

Reduced grain size of nanostructured ceramics leads to better mechanical properties in comparison to earlier ceramics.^40^ Zirconia based ceramics exhibit very good mechanical properties especially the TZP ceramics which exhibit flexural strength in the range from 900–1200 MPa and fracture toughness which ranges from 7 to 10 MPa m^1/2^.^41,42^ Transformation toughening leading to 3–5% increase in grain volume exhibiting compressive strength around crack tip which prevents crack propagation.^34,43^ A study by Silva *et al.*^44^ observed that sintering of nanostructured yttria-stabilized zirconia blocks at 1400C increased their flexural strength by 1020 MPa, Weibull Modulus by 13.1 and fracture toughness value by 11.2 MPa m^1/2^. Nanostructured ceramics exhibit the property of super elasticity which is 34 times faster for YSZ than the sub micrometer grained YSZ at a similar temperature.^45^ The deformation phenomenon of nanoceramics is related to dislocation motion or by sliding of grain boundary though a complicated coalescence of effects and relationships determines the super elastic behaviour.^46^

## 3. Mechanism of action

### 3.1. Antimicrobial action

**Fig. 1:**
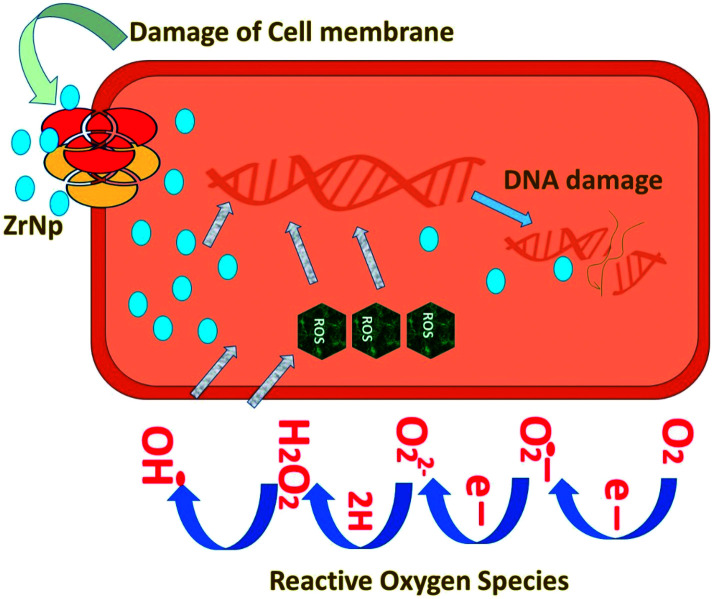
Antibacterial action of ZrNp.

**Fig. 2:**
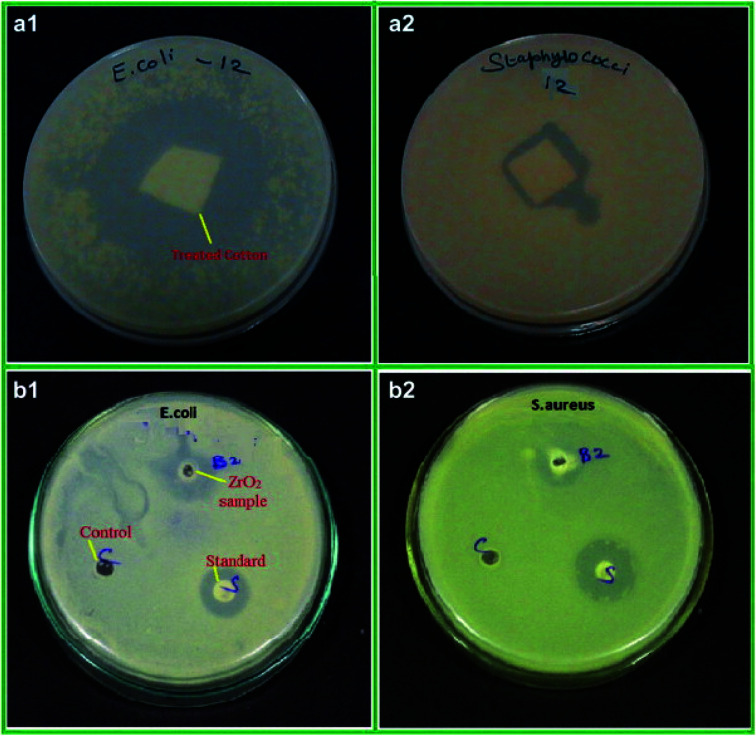
Antibacterial activity of ZrO_2_ nanoparticles treated cotton (a), ZrO_2_ nanoparticles (b) against *E. coli* (1) and *S. aureus* (2), respectively [this figure has been adapted/reproduced from ref. 51 with permission from Elsevier, copyright 2022].

ZrNp exhibit antibiofilm action so that these can be incorporated in biomaterials as antimicrobial agents^47^ (Fig. 1). The antimicrobial action of ZrNp is due to the formation of reactive oxygen species by ZrNp that inhibits growth of bacterial cells *Staphylococcus aureus* (*S. aureus*). It also causes disorganization of cell membranes *Escherichia coli* (*E. coli*) that leads to increase of permeability of membrane. This leads to accumulation of ZrNp in the cytoplasm causing cell damage. The raised ROS has an effect on lipid peroxidation that impacts the integrity of bacterial membrane causing membrane leakage.^48^ These ROS also terminates gene expression and causes damage to the DNA. In addition to arresting gene expression, ZrNp ions can produce denaturation of proteins by interruption of the metal ions within the metalloproteins. The released ions from the ZrNp can interrupt the metal cation cell stability that finally leads to death.^49^ Research has also shown that altering the surfaces characteristics of ZrNp can have an enhanced antibacterial effect against the biofilms. Altering the ZrNp surface with glutamic acid that consists of COO^−^ and NH^+^ ions assist ligand binding with ZrNp surfaces. This biofunctionalized ZrNp has a more stable negative charge that interact with rarely occurring positive clusters on cell wall of bacteria causing cell wall interaction and cell lysis.^50^ The dispersion of nanoparticles within the membrane of the bacteria is completely proportional to the nanoparticle size. The reduced size and increased surface area of ZrNp plays an important role in providing to aspects of surface energy as increased surface area is available for surface associated activities. ZrNp has shown to eliminate *E. coli* and *S. aureus* depicting its antibacterial spectrum due to action of active oxygen species and disorganization of cell membrane causing increased membrane permeability (Fig. 2).^51^ The lesser the size of nanoparticles, the greater the prospects of infiltrating and harming the bacteria membrane. The existence of transporter protein and ion channels facilitates the ZrNp nanoparticle movement across the membrane.^49^ Research done on particle size of ZrNp has shown that particle size of 4.8 nm showed an efficient activity against Gram positive and Gram negative bacterial strains.^52^ Another study showed that ZrNp of particle size in range of 9–11 nm showed scavenging effect by inhibiting free radicals exhibiting antioxidant effect.^53^ Thus, particle size in range of 4–11 nm of ZrNp can have a significant effect on bacterial cell growth. Spherical shape and smaller diameter size of ZrNp can also lead to increased antimicrobial action. Other possible action could be as explained by Jangra *et al.* in 2012.^54^ Results of this experiments were suggestive of crystal plane oriented antimicrobial action of ZrNp and its complexes. This could be due to different atomic arrangements of various exposed surface areas. Thus, different mechanisms have been put forth in relation to antimicrobial action of ZrNp.

### 3.2. Antifungal action

**Fig. 3:**
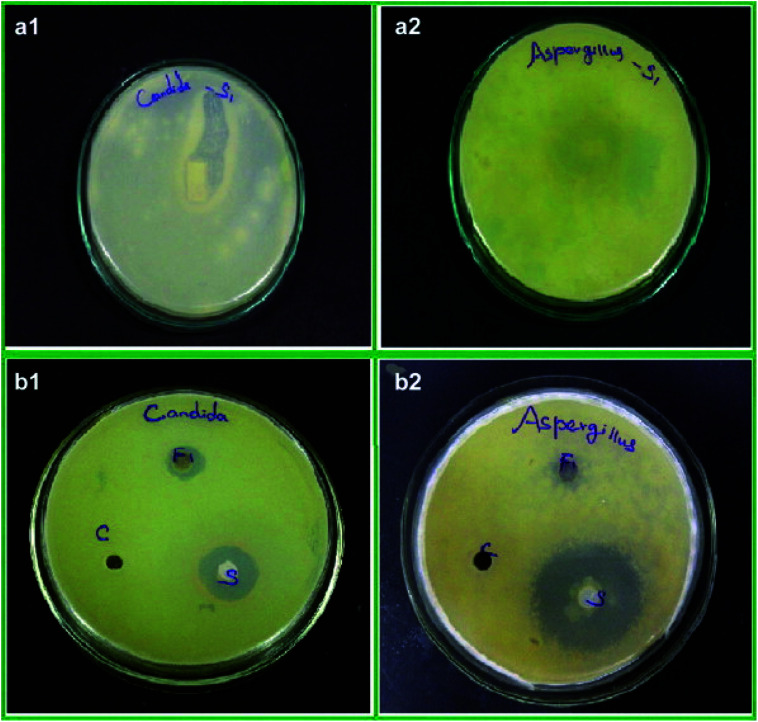
Antifungal activity of ZrO_2_ nanoparticles treated cotton (a), ZrO_2_ nanoparticles (b) against *C. albicans* (1) and *A. niger* (2), respectively [this figure has been adapted/reproduced from ref. 51 with permission from Elsevier, copyright 2022].

Due to high surface area of ZrNp, they exhibit significant antifungal action. Research have shown that they inhibit growth of *Aspergillus. niger* (Fig. 3)^54^ and *Candida . albicans*.^55^ The action is due to interference of cell functioning and deforming the fungal hyphae.^51^ Study has depicted that ZrNp fills up gaps of polymeric chain on the surface of a material. Due to appropriate bonding with polymer matrix, it leads to smooth surface that inhibits adhesion of *C. albicans*.^55^

## 4. Recent applications of ZrNp in dentistry

**Fig. 4:**
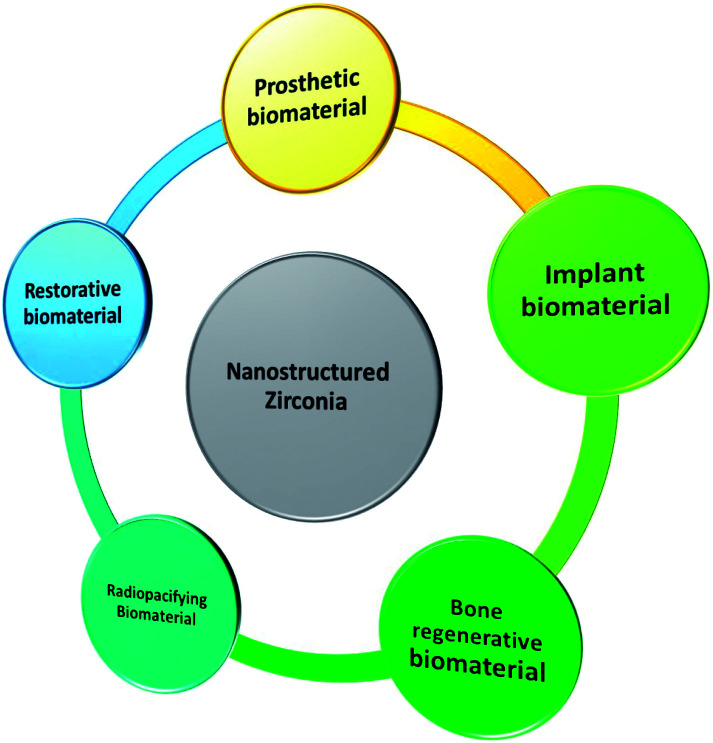
Recent applications of ZrNp as a biomaterial.

Recent applications of nanostructured zirconia have been graphically summarized in Fig. 4.

### 4.1. Restorative biomaterial

Chan *et al.* 2009 (ref. 56) investigated the effect of incorporating nanosized ZrO_2_ fillers in 3-component monomer solution (GTE) resin matrix on the fracture toughness of dental nanocomposites. The formulation of the composite resin is a GTE comprised of 37.5 wt% bis-GMA, 37.5 wt% bis-EMA and 25 wt% TEGDMA. The fillers are made up of 12 nm ZrNp with or without yttria dopant. Three-point bend test was used to determine elastic modulus, flexure strength and fracture toughness. The range of elastic modulus and flexure strength for the nanocomposites fell between 3.74–4.4 MPa and 71.3–106 MPa respectively. In terms of fracture toughness, ZrO_2_ nanocomposites with 10% yttria exhibited the highest value at 0.86 MPa m^1/2^. The fracture toughness of all the ZrO_2_ nanocomposites were statistically higher (*p* < 0.05) than the control, Z100 and the GTE resin. The better fracture toughness of the ZrO_2_ nanocomposites can be explained by the phenomenon where ZrO_2_ fillers deflect crack propagation in a tortuous path around the clumps of ZrO_2_ particles and along the matrix/particle interface. As the deflected crack moves along the particle/matrix interface, the higher fracture toughness of the ZrNp and the better interface bonding strength give rise to nanocomposites that are more resistant to fracture.

**Fig. 5:**
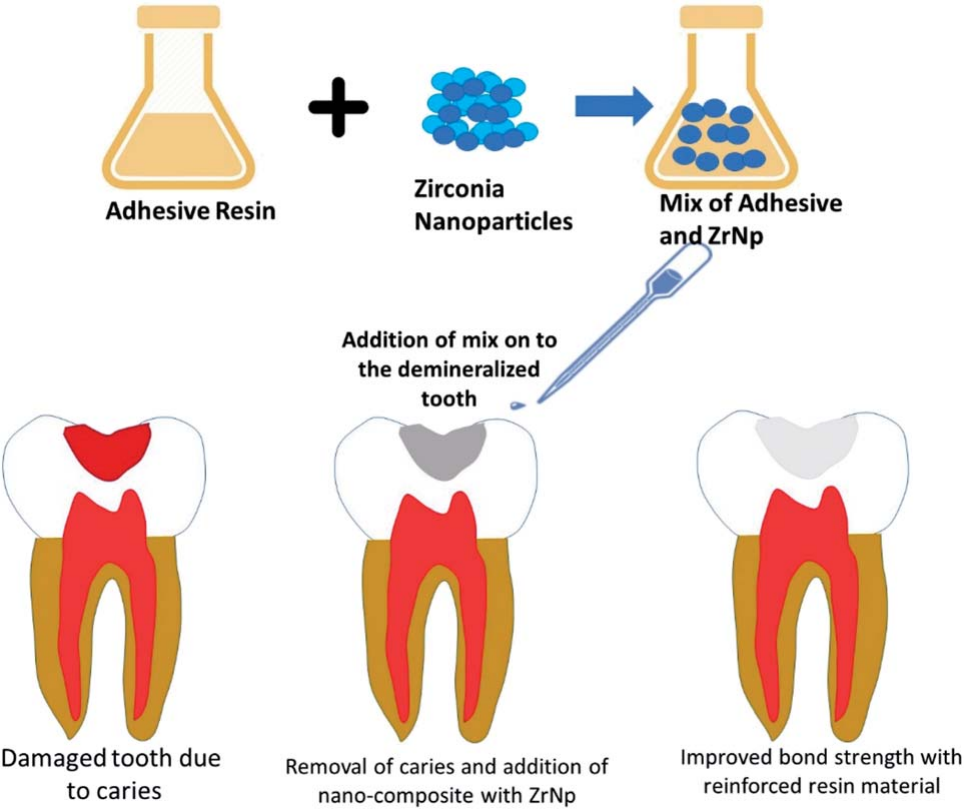
ZrNp prepared by CO_2_ laser vaporization added into composite resins improves the mechanical properties of the resin.

Lohbauer *et al.* 2010 (ref. 57) investigated how did the addition of zirconia nanofillers into the primer or adhesive resin component of the Scotch bond Multi-Purpose adhesive system (SBMP) affected the dentine bone strength, and the correlation with the morphological features at the bonded interface. The spherical ZrNp (20–50 nm) used in this study were produced with a CO_2_ laser evaporation method. These nanofillers were dispersed into the primer or adhesive solutions of the SBMP. The microtensile bond strength was measured, and the bonded interface examined under the scanning electron microscope and transmission electron microscope. The addition of ZrNp resulted in significantly higher microtensile bond strength than the control (*p* < 0.05) (Fig. 5). However, in the primer group, only 20% wt. filler content translated into significant bond strength increase. Most of the fractures in this study were caused by adhesive failures. Transmission Electron Microscopy (TEM) revealed that nanoparticles in the primer group were deposited on top of the hybrid layer, whereas in the adhesive group they are dispersed throughout. The nanoparticles incorporation had a reinforcing effect on the adhesive resin, hence increasing the bond strength. The nanofiller reinforced adhesives behaved like a barrier against easy crack propagation. Deviation of the crack path led to a greater crack length, resulting in higher energy demand for fracture to occur. It is also speculated that the local plastic deformation around the stiffer zirconia particles had a role to play. Moreover, the non-functionalized ZrNp reduced the tensile stress built up around them as the monomer was not rigidly affixed during polymerization. Furthermore, the infiltration of zirconia nanofillers between the dentinal tubules helped to form stronger resin tags. Martins *et al.* 2013 (ref. 58) investigated the radiopacity and microhardness of adhesives reinforced with varied concentration of ZrNp. By incorporating the ZrNp (20–30 nm) into the simplified adhesive system Ambar (with manufacturer's filler removed), 5 experimental adhesive resin systems were fabricated based on the filler weight percentage (wt%): 0% (EX0), 15% (EX15), 25%(EX25), 35%(EX35) and 50%(EX50). The digital radiopacity (% white) was determined by pixel counting and compared between the experimental adhesives, enamel, dentine, and a commercial adhesive (SB). All the experimental adhesives except for EX0 demonstrated radiopacity similar to enamel (*p* > 0.05) and greater than SB (*p* < 0.05). The microhardness of the experimental adhesives with filler loading of 25% and above was significantly higher than the commercial SB (*p* < 0.05). In the present study, 15% ZrNp were able to confer a level of radiopacity comparable to enamel. This was supported by Schulz *et al.* who found out that addition of agglomerated Ta_2_O_5_/SiO_2_ nanoparticles improved the radiopacity of adhesives. Filler loading with 25% or more of ZrNp contributed to higher microhardness, but this feature alone could not explain why the unfilled experimental adhesives (EX0) had greater microhardness than SB. It is plausible that a more balanced and adequate monomer composition blend in EX0 resulted in a polymeric material with improved mechanical properties. Therefore, incorporating ZrNp into adhesives can be an option to produce a radiopaque restorative material with improved microhardness. Badr 2018 (ref. 59) compared the physical and mechanical characteristics of light cured composite modified with varying concentration of ZrNp. The composition of the composite resin used in this study was ZrNp (<100 nm), barium borosilicate glass filler (9.5 μm), γ-MPS, Bis-GMA and TEGDMA. 5 experimental groups were fabricated according to the weight percentage (wt%) of the ZrNp (1, 3, 5, 7 and 10%). The properties measured included water sorption, solubility, volumetric changes, depth of cure, flexural strength, elastic modulus and diametral tensile strength. The water sorption, solubility and volume increases followed an upward trend with increasing concentration of ZrNp, with the highest values obtained for 10 wt% composite. The same composite group also exhibited the lowest depth of cure at 2.3 mm. The 1 wt% group displayed the highest flexural strength and diametral tensile strength, while the 5 wt% group possessed the highest flexural modulus. The water sorption and solubility were greater at higher concentration of ZrNp because of the bigger surface area afforded by the nanoparticles. In terms of water sorption, the greater surface area facilitated water diffusion between the nanoparticles and the polymer. Since there was more surface area for removal of non-polymerized fillers, the water uptake increased, causing the composite to swell, and increased in volume. The depth of cure was adversely affected by increasing the concentration of ZrNp due to their high density and smaller filler size, which scattered and attenuated the curing light during the polymerization process. It was worth noting, however, that despite the impact on water sorption, solubility and depth of cure, the values still abided by the ISO 4049 standard. The incorporation of small ZrO_2_ nanoparticles at 1 wt% led to improved flexural strength, but it subsequently dropped at higher concentration. The same trend was observed for diametral tensile strength. This might be due to the poor interfacial bond between the particles and resin matrix, as there was insufficient resin matrix to bond to both the nanoparticles and the glass fillers. The combination of the glass and ZrO_2_ filler particles explained why there was a higher flexural modulus for the 5 wt% group. Rodriguez & Casanova 2018 (ref. 60) investigated the roughness and nanohardness of dental composite resin reinforced with either nanoparticles or nanoclusters. The nanoparticles in this study were made up of silica in an aqueous dispersion (20 wt% of nanoparticles) and/or zirconia in an aqueous dispersion (30 wt% of nanoparticles). The monomer composition included Bis-GMA, Bis-EMA, UDMA and TEGDMA. The silica and silica-zirconia nanoclusters were obtained by spray drying. Roughness of the non-aggregated silica nanoparticles was significantly lower than the 2 nanoclusters (*p* < 0.05). All three materials in this study exhibited nanohardness values between 0.2 to 0.25 GPa, and the difference between groups was not significant (*p* > 0.05). Non-aggregated silica nanoparticles exhibited the smoothest surface by virtue of its smaller particle size, bigger surface area available for bonding, and formation of strong chemical bond to the polymeric matrix. The inclusion of zirconium dioxide, which was not functionalized, compromised the bond strength. This might explain why the silica-zirconia nanoclusters shared similar roughness with its silica counterpart despite having lower particle size. Another explanation for the poor performance of nanoclusters in the roughness test is the lack of chemical bonding between the nanoparticles. As a result, the nanoparticles can be dislodged from the material with relative ease, hence imparting a rougher surface. The nanohardness values for all 3 materials were very identical, because they have similar filler concentration (20–30% by weight). The particle size and presence/absence of functionalization had no appreciable effect on the average hardness of the composite (Table 2).

**Table 2:** Application of ZrNp as restorative biomaterial

Author/year	Application	Type of nanoparticle	Objective	Method of preparation	Particle size (nm)	Outcome	Ref.
Chan *et al.* 2009	Nanocomposites	ZrNp with or without yttria content	To assess if using ZrO_2_ particles in nanocomposites improves its mechanical properties (fracture toughness, flexure strength and elastic modulus)	Flame spray pyrolysis	12	Improved fracture toughness of nanocomposites	56
Lohbauer *et al.*, 2010	Adhesive system	Spherical ZrNp	To assess if inclusion of ZrNp into the primer or adhesive resin give rise to better dentine bond strength	Laser evaporation method	20–50 nm	Enhanced microtensile bond strength to dentine	57
Martins *et al.*, 2013	Adhesive system	ZrNp	To evaluate the effect of adding ZrNp on the radiopacity and microhardness of an experimental adhesive system	Using a simplified adhesive system Ambar as the base, the ZrNp were silanized, dried and disaggregated in a pistil	20–30 nm	Improved radiopacity that can match enamel, and better microhardness	58
Badr *et al.*, 2018	Composite reins	ZrNp	To evaluate and compare the mechanical and physical characteristics composites incorporated with varying concentration of ZrO_2_ nanoparticles	Dispersion of ZrO_2_ nanoparticles into a Bis-GMA/TEGDMA resin with glass fillers up to 76 wt%	<100 nm	Increasing the concentration of ZrO_2_ nanoparticles increases the water sorption, water solubility, volumetric increase and flexural modulus (up to 5 wt%) but decreases the depth of cure. Flexural strength and diametral tensile strength of composite can be improved by addition of these nanoparticles, but only in small amount (1% weight by percentage)	59
Rodríguez *et al.*, 2018	Composites	(1) silica nanoparticles	To assess the roughness and nanohardness of dental composites reinforced with nanoparticles and nanoclusters	Nanoclusters prepared by spray drying	(1) 40–60 nm	Silica nanoparticles displayed lower roughness, but similar hardness compared to the nanoclusters	60
		(2) ZrNp			(2) 5–10 nm		
		(3) silica nanoclusters			(3) 4.5 μm		
		(4) silica-zirconia nanoclusters			(4) 2 μm		

ZrNp are not only able to increase certain mechanical properties of both composite resin and adhesive system, but also exerts antibacterial action, which could be important to prevent secondary caries formation, one of the most common causes of failure for conventional composite system. However, it is prudent to investigate whether the increased water sorption, water solubility and decreased depth of cure will lead to compromises in the clinical performance.

### 4.2. Prosthetic biomaterial

**Fig. 6:**
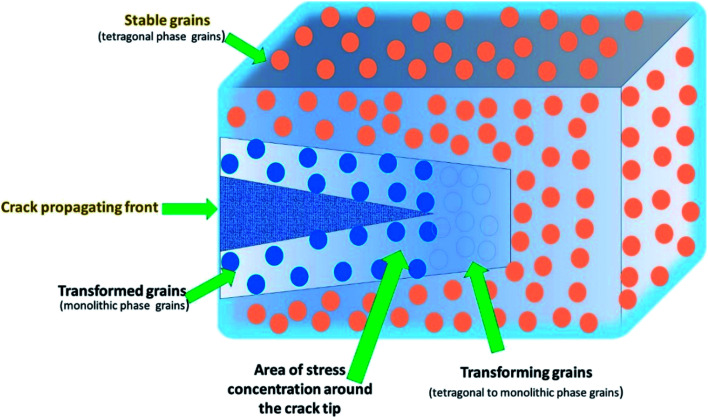
Transformation toughening mechanism of ZrNp.

A remarkable development in dentistry has been the introduction of ceramics based on zirconia with its mechanical features mostly associated with the phase transformation from tetragonal to monoclinic (t → m)^61^ (Fig. 6). Many new types of zirconia ceramics have been used these days including the ceria-stabilized tetragonal zirconia/alumina nanocomposite, In-Ceram Zirconia (IZ), DC-Zirkon (DZ), diatomite-based nanocomposite ceramics, hybrid ceramics of nano-HA/ZrNp (Table 3). Guazzato *et al.* 2004 (ref. 61) evaluated the microstructure, strength and fracture toughness of experimental yttria partially stabilized zirconia, in-ceram zirconia slip, DC Zirkon and In-Ceram dry pressed. He observed that in IZ the mechanism of toughening can be attributed to following factors, like the contact shielding, crack deflection because of alumina grains, and microcrack nucleation, phase transformation because of zirconia particles. Among YZ (Y_2_O_3_ TZP) and DZ the variation of fracture toughness and strength is an attribution of the disparity in the grain size, amount of stabilizing oxide (yttria), processing method used and their effect on the porosity and metastability of tetragonal grains. Philipp *et al.* 2010 (ref. 62) evaluated the clinical functioning of veneered ceria-stabilized zirconia/alumina nanocomposite frameworks used for posterior three-unit Fixed Partial Dentures (FPD). It was found that this foundation material was clinically dependable with a 100% survival rate. There was no damage or breakage of the veneering ceramic, and the biologic outcome of the FPDs' was good but studies with extended observation period and a larger sample size are needed. Lu *et al.* 2012 (ref. 63) fabricated high strength diatomite based ceramics using layer-by-layer (LBL) technique for covering diatomite particles by polymers to enhance the adsorption and dispersion of positively charged ZrNp. It was found that there was reduction of the particle size and narrowing of the size distribution range of diatomite powders. The surface of diatomite-based powder got negatively charged leading to adsorption of positively charged ZrNp leading to enhancement of the sintered ceramics. The adsorbed ZrNp form a protective barrier preventing crack extension and lead to crack deflection, all of which improved the mechanical properties and increased shear bond strength.

**Table 3:** Application of ZrNp as prosthetic biomaterial

Author/year	Application	Type of zirconia	Objective	Method of preparation	Particle size (nm)	Outcome	Ref.
Guazzato *et al.*, 2003	All ceramic restorations	Experimental yttria partially stabilized zirconia, DC Zirkon, in-ceram zirconia slip and in-ceram dry pressed	To assess the fracture, strength toughness and microstructure, of four types of ceramics	NA	NA	Zirconia based dental ceramics exhibited better mechanical properties as compared to the conventional glass-ceramics	61
Philipp *et al.* 2010	Veneers, fixed dental prostheses	Ceria-stabilized tetragonal zirconia/alumina-nanocomposite, zirconia veneering ceramic (Vintage ZR, Shofu)	To evaluate the clinical performance of veneered frameworks for posterior three-unit FDPs made from Ce-TZP/A nanocomposite	CAD/CAM system (Hint-Els)	—	After 1 year of use Ce-TZP/A nanocomposite framework was found to be sound and dependable	62
Lu *et al.* 2012	Veneers	Diatomite-based nanocomposite	To manufacture high strength diatomite-based ceramics for use in dental applications	Diatomite particles were coated with anionic and cationic polymers using layer by layer technique and then sintered with nano-ZrO_2_	—	Adding 30 wt% nano-ZrO_2_ lead to enhanced mechanical characteristics (fracture toughness and flexural strength) along with decreased porosity. The shear bond strength of diatomite-veneer ceramic was also increased	63
Lian *et al.* 2018	Medical implants	Hybrid nano-ceramics of HA-ZrO2	To improve the crystalline size, mechanical properties and cytocompatibility of nano-HA ceramics by sintering with nano-ZrO_2_ to form hybrid ceramic	Two step sintering method was used prepare hybrid bioceramics of nano-HA and nano-ZrO_2_	—	Hybrid nano ceramics exhibited decreased elastic modulus, hardness and crystallite size and similar cytocompatibility to nano-HA ceramic	64
Park *et al.* 2013	Prosthetics	ZrNp	To consolidate ZrO_2_ nanopowders using MPC to enhance them to be used as ZrO_2_ dental blocks	Magnetic pulsed compaction (MPC) and two-step sintering		MPC resulted in the development of fully dense (∼98%) ZrO_2_ bulks. Under optimum processing conditions the bulk produced had good formability	65

Lian *et al.* 2013 (ref. 64) studied the effect of addition of ZrNp into nano-HA ceramics to improve the crystallite size, mechanical properties and cytocompatibility of hybrid ceramics. It was found that the mechanical properties had enhanced as evidenced by reduced elastic modulus and crystallite size along with similar cytocompatibility of hybrid ceramics to MC3T3-E1 cells. The reduction is crystallite growth is due grain boundary pinning of ZrNp diffused in around the nano-HA lattice inhibiting the particle boundary movement along with triple drag point. The lower alter degree of crystallite dimension results in increased fracture strength with reduced elastic modulus and hardness. Park *et al*. 2013 (ref. 65) consolidated ZrNp using magnetic pulsed compaction and evaluated the outcome of blending conditions, magnetic pulsed compaction pressure and sintering temperature on the hardness, shrinkage, machinability and density to enhance them to be used as ZrO_2_ dental blocks. MPC resulted in the development of fully dense (∼98%) ZrO_2_ bulks. Under optimum processing conditions (MPC1GPa, 1.0 wt% PVA) the bulk produced had fair hardness (1150 Hv), ∼19% shrinkage, ∼97% density and good formability. There was reduction in the grain size with increase in magnetic pulsed compaction (MPC) pressure but the proportion of PVA had less outcome on grain size. Marefati *et al.* 2018 (ref. 66) investigated the wetting action of molten feldspathic glass-ceramic for veneering dental Yttria-stabilized Tetragonal Zirconia Polycrystal cores. There was activation of chemical wetting mechanism leading to decrease in contact angle with time at 990 C for prolonged period. The wettability was affected by the grain size of Yttria-stabilized Tetragonal Zirconia Polycrystal with the mean contact angle getting reduced by 6% for nano sized grains in comparison to micronized grains. Firing time is reduced to get better wettability with nanosized zirconia as compared to micronized zirconia. Daud *et al.* 2017 (ref. 67) evaluated and analyzed the shear bond strength (SBS) of different core materials with two veneering porcelains and also observed the manner of bonding failure. The experimental zirconia had similar results with commercially obtainable zirconia when used with same veneering porcelain. Zirconia made using slip casting or compaction method had almost identical bonding abilities to veneering porcelain. The bond strength between core and veneer is material dependent. A mild positive Coefficient of Thermal Expansion (CTE) mismatch between the framework and the veneer leads to superior bond strength and a negative CTE variance result in low SBS values. Cohesive failure is observed in combinations with higher SBS values while mixed adhesive and cohesive failure is there in those with lower SBS values.

Preliminary findings indicated that nanostructured zirconia can be used to fabricate fixed dental prothesis with enhanced properties. However, inherent challenges in maintaining high densities of nanocrystalline ceramics remain, and the outcome from using innovative techniques such as magnetic pulsed compaction should be further explored.

### 4.3. Implant biomaterial

Titanium and its alloy are widely used in dental implant construction. Some of the properties that make them the dental implant material of choice include good mechanical strength, formability, corrosion resistance and biocompatibility.^68–75^ The most ubiquitous form of titanium in use today are the alpha beta titanium alloy Ti–6Al–4V and commercially pure titanium.^76^ Despite a generally favorable biocompatibility, Ti–6Al–4V had been implicated to possess cytotoxicity due to the release of aluminum and vanadium ions.^77^ Furthermore, the elastic modulus of these conventional implant materials exceeds that of natural bone. This discrepancy in modulus of elasticity can cause stress shielding and bone resorption, eventually culminating in implant failure.^78^ Therefore, there is a growing need for alternative materials or alloy combination that will produce better clinical outcomes. ZrNp, with its excellent biocompatibility, osteoconductivity, soft tissue stability and esthetic appeal, can be a promising alternative.^79^ Besides the material composition, surface topography of the implant is a critical factor in optimal osseointegration as well. Currently, researchers are looking into leveraging nanotechnology for implant surface modification. This review article aimed to provide a comprehensive overview of the application of ZrNp patterning in implant dentistry (Table 4).

**Table 4:** Application of ZrNp as Implant biomaterial

Author/year	Application	Type of zirconia	Objective	Method of preparation	Particle size (nm)	Outcome	Ref.
Lucas *et al.* 2015	Prosthetic aspects in implant dentistry	Yttria-tetragonal zirconia polycrystals (Y-TZP)	To assess the impact of grain size on phase transformation (tetragonal to monoclinic) induced by artificial aging, and accompanying changes in surface roughness, modulus of elasticity and nanohardness	Supplied in post-sintered polished state	350–574 (grain size)	Smaller grain size of Y-TZP was associated with a higher percentage of monoclinic transformation, but similar surface roughness, modulus and nanohardness post aging	127
Minagar *et al.* 2015	Implant dentistry	Titania-zirconia-zirconium titanate (TiO_2_–ZrO_2_–ZrTiO_4_)	To investigate the effect of TiO_2_–ZrO_2_–ZrTiO_4_'s dimensional properties (nanotube size, surface roughness and surface energy) on the osteoblast cellular responses	Anodization by different applied potential	76–108	The osteoblasts on the surface of nanotubes with inner diameter of 40 nm presented with the highest density and longest filopodia. Maximum surface roughness was found on 59 nm wide nanotubes	84
Oshima *et al.* 2017	Implant dentistry	Ceria tetragonal zirconia polycrystal based zirconia/alumina (Ce-TZP/Al_2_O_3_) nanostructures	To study the osteoblast cellular response and osseointegration potential of Ce-TZP/Al_2_O_3_ modified by hydrofluoric acid	Mechanical preparation of Ce-TZP/Al_2_O_3_ specimen into disk using forming surface grinder	300–500 nm	After HF treatment, the Ce-TZP/Al_2_O_3_ surface developed a greater surface area, roughness and geographic undercut, as well as a more marked osteoblastic response and better osseointegration	80
Soon *et al.* 2017	Implant dentistry	Yttria-stabilized zirconia/gadolinium doped ceria (YTZ/GDC) nanoislands	To evaluate the difference in osteoblast behaviour when the substrate surface is modified by nanoislands	Thermal annealing of YTZ with GDC by powder-suspension based method	—	Nanopatterned substrates with YTZ/GDC induced favourable biologic response of the osteoblasts by enhancing spreading, growth, differentiation and mineralization	128
Blackert *et al.* 2018	Implant dentistry	Titanium–niobium–zirconium–Tantalum (Ti–35Nb–7Zr–5Ta)	To analyse the microstructure, morphology and composition of TNZT alloy and its nano-scaffolds after undergoing hydrothermal treatment	TNZT alloy ingot manufactured by vacuum arc melting and subjected to hydrothermal treatment with sodium hydroxide solution	Average grain size of 50 μm for TNZT alloy; 280–370 nm for the nano scaffold oxide layer	Hydrothermal treatment of TNZT sheets with 5.0 M sodium hydroxide gave rise to titanium oxide rich nano scaffold (average pore size 60–80 nm) that bode well for bioactivity and osseointegration	81
Rezaei *et al.* 2018	Implant dentistry	Yttria-stabilized tetragonal zirconia polycrystal (Y-TZP)	To compare the biological behaviour and osseointegration capacity of hierarchically roughened zirconia and machined smooth zirconia	Solid state laser etching to create roughened zirconia	100–400 nm	Hierarchically rough zirconia resulted in better osseointegration than machined smooth zirconia owing to better osteoblasts differentiation without compromising the cellular proliferation	88
Gnilitskyi *et al.* 2019	Implant dentistry	TiO_2,_ Al_2_O_3_, Al(OH)_3_, ZrO_2_	To assess the biological compatibility of the Ti6Al4V and Zr implant surfaces nanotextured by femtosecond laser	Highly regular laser induced periodic surface structures method	800 nm for Zr; 820 nm for Ti	Laser nanostructured surfaces demonstrated better cell adhesion and proliferation compared to untreated surfaces, while the composition (Ti alloy or Zr) played a less substantial role	82
Xiao-Feng *et al.* 2011	Implant dentistry	Nano hydroxyapatite, ZrO_2_	To determine the characteristics of the nano-HA/ZrO_2_ composite surface coating on the titanium alloy	Electrochemical method	—	Under the correct conditions (electric current, electrodeposition time), a uniform composite coating of nano-HA and ZrO_2_ with a higher combined strength can be formulated	126
Pang and Huang, 2012	Implant dentistry	Nano hydroxyapatite, ZrO_2_	To develop an understanding of the physical properties and biological compatibility of a nanohydroxyapatite/ZrO_2_ composite coating on the surface of titanium material	Electrochemical method	10 μm	The nano HA/ZrO_2_ composite coating developed in this study had excellent combined strength, good biocompatibility and was biologically active	86

Oshima *et al.* 2017 (ref. 80) investigated the ability of nanostructured zirconia/alumina composite(Ce-TZP/Al_2_O_3_) treated with hydrofluoric acid (HF) to osseointigrate. An *in vitro* component of this investigation consisted of culturing mouse osteoblastic cells on acid etched Ti disks and Ce-TZP/Al_2_O_3_ disks without HF treatment and 4%, 55% HF treated disks and assessment of the biological response. Whereas an *in vivo* part consisted of placement of miniature implants made up of Ti and 55% HF treated Ce-TZP/Al_2_O_3_ in rat femora for the assessment of osseointegration. 55% HF treated disks showed highest cell attachment and proliferation after 1 day and 5 days incubation period. Similar results were observed with respect osteoblast differentiation. The push in test value showed favorable osseointegration with 55% HF treated Ce-TZP/Al_2_O_3_ group. The presence of nano structured Al_2_O_3_ particles which are HF resistant in Ce-TZP/Al_2_O_3_ have the osteogenic induction property leading to increased ALP activity and bone related gene expression. Unlike to other studies 55% HF treated Ce-TZP/Al_2_O_3_ promoted osteoblastic proliferation as well as differentiation as owing to the nano architecture caused due to HF treatment which could be applied in bone regeneration and engineering. Same HF treatment led to increased undercuts and increased surface roughness. All these features could be the possible reasons for the 1.6-fold increased osseointegration strength. Thus 55% HF treated Ce-TZP/Al_2_O_3_ could be considered as a potential novel implant material. Blackert *et al.*, 2018 (ref. 81) aimed to investigate the physical and chemical attributes of nano-TNZT (Ti–35Nb–7Zr–5Ta-titanium based alloy with reducing quantity of Nb, Zr, Ta). Vacuum arc melting was used to produce TNZT alloy and immersed in sodium hydroxide solution followed by assessment of physical and chemical properties. Higher concentration and more time of immersion of sodium hydroxide led to significant amount of nano scaffold formation with increased pore size along with significantly higher O/Ti ratio. Under scanning electron microscopy (SEM) cracking was observed only in those samples which were exposed to higher concentration of sodium hydroxide. A threefold increase of the relatively smooth compact oxide layer was observed on addition of TiO_2_ nanoparticles to the hydrothermal solution. Sodium hydroxide concentration was the principal factor affecting Raman spectra and higher concentrations led to increased Ti-rich oxide layer. Longer time of hydrothermal processing led to more etching and increased pore size. Cracking could be attributed to thermal contraction caused by the difference in the coefficient of thermal expansion. Larger amount of OH^−^ radicals in higher concentration of sodium hydroxide oxidized elemental titanium.

Gnilitskyi *et al.* 2019 (ref. 82) investigated and compared the effects of femtosecond laser nanotexturing on Ti6Al4V and Zr implant surfaces in context with their biological compatibility. Polished samples of commercial grade-5 titanium alloy (Ti6Al4V) and zirconium (Zr, 99.7% purity) underwent nano structuring using HR-LIPSS which combines micron-scale Low Spatial Frequency LIPSS (LSFL) with nano-scale roughness highly effective for osteointegration processes and assessed for surface characterization and contact angle measurement. Samples with modified and non-modified surfaces were incubated with HDFa (Human Dermal Fibroblasts-Adult) and cell viability was measured in the *in vitro* part of this investigation. *In vivo* part included implantation of modified and nonmodified surfaces of both titanium (Ti) alloy and Zirconia (Zr) in rat model and carefully removed after 30 days to prevent damage of the tissues that were covering implants, processed and observed under SEM to measure the presence of cells and fibers on the surface of the sample and their distribution, cells density per cubic mm, and the fibers size. Homogeneously speeded, regular nanostructures were observed under SEM on the modified surfaces of both the groups. X-ray photoelectron spectroscopy (XPS) revealed the presence of hydroxides and carbonaceous species on the surface. The Highly Regular LIPSS (HR-LIPSS) did not alter the hydrophilic nature of both the surfaces, but cell viability, attachment, and proliferation was significantly improved, with no statistical intergroup differences. On 10th day, both groups of modified implants were covered by thin capsule of connective fibers and erythrocytes, fibroblasts, and leucocytes, with density of fibroblasts more on Zr surfaces. The control group lacked all such features. After the 30 days of implantation, treated metal implants could hardly be removed as they were completely integrated with surrounding tissues and completely covered with connective tissue-like structures with high number of fibers. Hydroxides and carbonaceous species may hasten the adsorption of proteins on the implant surface accelerating implant healing and increasing the implant surface. Nano topography may positively affect early, fast and complete protein adhesion upregulating the integrin-mediated cellular cascade thus accelerating cell attachment and proliferation. Cell proliferation appeared to be independent of the type of metal but highly influenced by the surface topography and surface oxidation and HR-LIPSS could be a novel method for it.

**Fig. 7:**
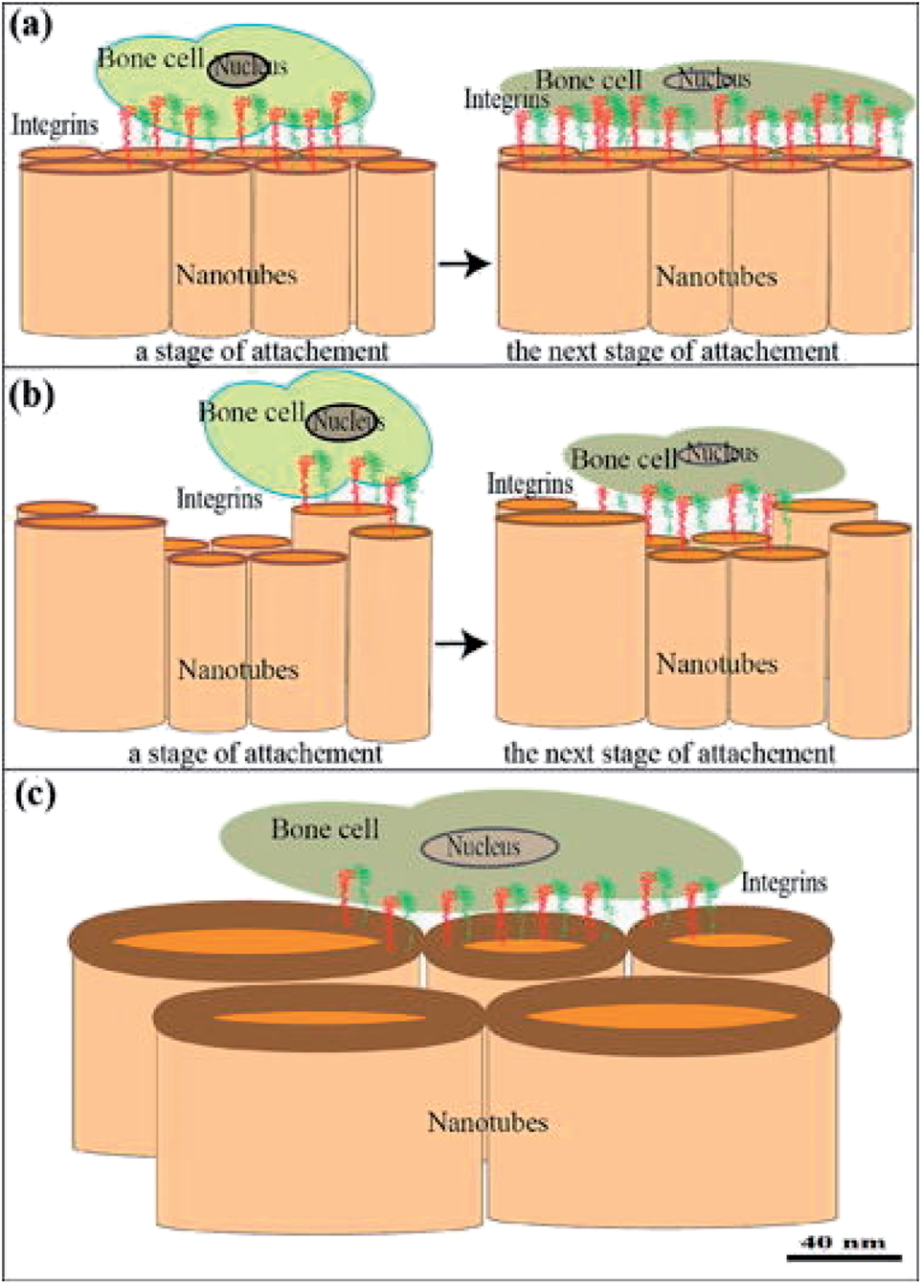
(a–c) Cell attachment to nanotubes showing early and later phases of adhesion with cell surface receptors and integrins. They transmit a signal from the ECM to the nucleus to regulate many cellular activities. Communication with the ECM-adsorbed biomolecules between tissue cultured cells and the substrate surface [this figure has been adapted/reproduced from ref. 84 with permission from Elsevier, copyright 2022].

At the present, a plethora of mechanical, physical and chemical modification techniques exist for dental implant surfaces, such as sand blasting, coating, acid etching and thermal processing.^83^ Anodization, an electrochemical surface treatment was used in a previous study to develop TiO_2_–ZrO_2_–ZrTiO_4_ nanotubes on the surfaces of titanium zirconium alloy. It was found that these nanotubes were able to enhance the adhesion of osteoblast, with the greatest cell density on nanotubes with inner diameter of 40 nm (Fig. 7).^84^ A composite coating of hydroxyapatite and zirconia was similarly produced by an electrochemical method. Microscopically, this unique surface pattern exhibited a remarkable combined strength of 17 GPa, which indicated that the ZrNp were successfully integrated in between the hydroxyapatite and titanium surfaces.^85^ A different study on HA/ZrO_2_ similarly reported positive outcomes of this composite coating both mechanically and biologically.^86^ Hydrofluoric acid treatment had also been shown to increase the surface area and roughness of zirconia. The resultant nanostructured Ce-TZP/Al_2_O_3_ stimulated the osteoblastic cell response and promoted better osseointegration.^80^ Soon *et al.* 2017 presented a simple and low-cost method of creating yttria-stabilized zirconia/gadolinium-doped ceria nanoislands on miscut substrate by thermal annealing.^87^ Another study had successfully grown Ti–35Nb–7Zr–5Ta nano scaffolds on TNZT *via* hydrothermal treatment with sodium hydroxide solution.^81^ A novel, sophisticated method was reported in the literature, harnessing solid state laser sculpting to a generate a hierarchically roughened zirconia surface at the meso-, micro- and nanoscale. This experimental approach yielded better integration at the bone-implant interface by expediting osteogenic differentiation without compromising the cellular proliferation.^88^ This finding was corroborated by another group of researchers that generate nanotextured implant surfaces by femtosecond laser. They even suggested that surface topography, and not composition (titanium or zirconia) played a more substantial role in determining the degree of osseointegration.^82^ Despite the variability of these techniques, they share one thing in common: osteoblastic cell activity and osseointegration were amplified by complex surface topography at the micro or nanostructural level.

It is undeniable that Zr implant holds great promise, not only as implant body material, but also its ability to modify the implant surface topography to enhance osseointegration. It is expected that Zr implant will be more extensively researched in the implant dentistry community in the continuous effort to come up with a material that can surpass the gold standard micro-rough titanium implants. Before that can come to pass, a priority is to evaluate the long term cytocompatibility of Zr implants.

### 4.4. Bone regenerative biomaterial

Research in bone tissue engineering is growing at a rapid pace for finding a substitute for conventional grafts for bone regeneration. The conventional grafting procedures pose some limitation such as increased cost, shortage of donors, transmission of diseases and repetitive surgery. Scaffolds constructed from biomaterials which mimic bone could be an alternative for such grafts.^89^ Pattnaik *et al.*, 2011 (ref. 90) fabricated a scaffold (bio-composite) of chitosan, silica and zirconia and assessed the characteristics of composite scaffold for its applications in bone tissue engineering. They also evaluated the cytotoxicity of the scaffold using osteoprogenitor cells of Wister rats. According to their observation, scaffold depicted suitability for infiltration of cells. Scaffold depicted biomineralization characteristics due to zirconia. It was found to be biocompatible to rat osteoprogenitor cells. Sang-Hyun An *et al.*, 2012 (ref. 91) investigated porous Zirconia/Hydroxyapatite (ZrO_2_/HAp) composite material for physical properties, cellular compatibility and its effect on the repair of bone tissue. They observed that ZrO_2_/HAp scaffolds fabricated showed superior mechanical properties with a higher degree of cell affinity without degradation. The scaffolds characteristics can be modified by altering the composition of ZrO_2_/HAp. These scaffolds possess vastly connected pores and thus can act as substrate for regeneration and reconstruction of various bone defects. By adjusting the composition of the ZrO_2_/HAp scaffold starting material, the characteristics of these scaffolds can be modified. These ZrO_2_/HAp scaffolds have a highly connected pores could be a promising substrate for obtaining the dual effect of both reconstruction and regeneration which is necessary for bone tissue repair of large bone defects. Afzal A 2014 (ref. 92) stated that zirconia depicted finest combinations of biocompatibility, fracture toughness, mechanical strength and thus can find its use in multiple applications as surface coating material of implants, as an scaffold, implant material and as a radiopacifying agent. Zhu *et al.*, 2015 (ref. 93) fabricated nano-sized zirconia scaffolds and studied cell proliferation, adhesion, pore size and porosity of these scaffolds. The results depicted that these scaffolds have promising properties at a porosity of 75.2% that can be used for bone regeneration. Deepthi S *et al.* 2016,^94^ provided a review of applications and characteristics of the Chitin and chitosan based nanoceramic composites. They also observed that nanocomposites formed an osseointegrating surface with the native bone which is of utmost importance in regenerating bone. Chitin/chitosan provides a suitable niche for the loading of cells as it is an extracellular mimicking biopolymer. Nanoceramics could be used as vehicles for delivery of antibacterial drugs and bone regeneration growth factors like BMP-2, FGF-18 due to their higher loading capacity. Aboushelib MN *et al.* 2017 (ref. 95) evaluated osteogenic ability of porous zirconia scaffolds (CAD/CAM deigned) which were added with hydroxyapatite for augmenting surgically created large bony defects in Beagle dogs. A computer software was utilized to perform the histomorphometry analysis of the bone-scaffold interface for detection of the amount of new bone formation. They concluded that the volume of new bone formation was significantly higher with hydroxyapatite enriched zirconia scaffolds as compared to controls. The pore cavity walls were the first site for deposition of new bone which then proceeded by filling the entire pore volume. Additionally, it was observed that mineralized bone matrix had islands of entrapped hydroxyapatite particles within it. Balagangadharan K *et al.* 2018 (ref. 96) characterized biocomposite scaffolds (CS/nHAp/nZrO_2_) composed of nano-zirconium dioxide (nZrO_2_), nano-hydroxyapatite (nHAp), chitosan (CS) which were manufactured using freeze–drying method along with microRNA (miRNA) for bone tissue engineering applications. Osteoblast differentiation at the cellular and molecular levels was evaluated by testing the outcome of a bioactive molecule (miR-590-5p) with scaffolds using mouse mesenchymal stem cells. Results showed that scaffolds supported differentiation of osteoblasts that was improved with miR-590-5p. Thus, scaffolds CS/nHAp/nZrO_2_ along with miR-590-5p can be applied for regeneration of bony defects.

Natural and synthetic polymers are utilized in the fabrication of biocomposite polymers. The osteogenic capacity of the scaffolds is greatly enhanced by the incorporation of metal oxides. Zirconium *via* the BMP/SMAD signalling pathway enhances the osteoblast proliferation acting as an osteoinductive material. Additionally, it also improves the mechanical properties of the scaffolds. Hydroxyapatite (HAp) along with ZrO_2_ improves its mechanical properties for its use in load bearing areas (Table 5).

**Table 5:** Application of ZrNp as bone regenerative biomaterial

Author/Year	Application	Type of nanoparticle	Objective	Method of preparation	Particle size (in nm)	Outcome	Ref.
Aboushelib MN *et al.* 2017	Bone augmentation	hHydroxyapatite was utilized to enrich the porous CAD/CAM zirconia scaffolds	Computer software was used to perform histomorphometric analysis to observe quantity of new bone development at the interface between bone and scaffold	Sintering of CAD/CAM zirconia blocks was perfromed to get the suitable shape which was followed by filling the scaffolds with a nano-hydroxyapatite powder	25–55 nm	The hydroxyapatite enriched zirconia scaffolds showed enhanced bone quantity compared to the controls. Coating of the pore cavity walls was the starting point for new bone deposition, and it continued by fill-up of whole pore volume. Mineralized bone matrix showed particles of hydroxyapatite formation	95
Gaihre B *et al.* 2018	Bone regeneration	Scaffolds of chitosan were hybridized with nano-calcium zirconate (CS-nCZ), nano-zirconia (CS-nZrO) and nano-hydroxyapatite (CS-nHA)	To compare the physical and biological properties of these three scaffolds	Conventional freeze-drying technique	nZrO (22–50 nm), nCZ (7–25 nm), and nHA (30–90 nm)	The physical properties were comparable among the three scaffolds, but significantly higher compared to CS alone scaffolds. Zirconia based bioceramic materials had a lower response of osteoblasts. CS-nCZ had an increased rate of growth of pre-osteoblasts in comparison with CS-nZrO	129
Balagangadharan K *et al.* 2018	Bone regeneration	The composition of scaffolds was – (1) nano-hydroxyapatite (nHAp), (2) ZrNp (3) chitosan (CS)	Evaluation of the additive impact of scaffolds of CS/nHAp/nZrO2 along with miR-590-5p on osteogenic potential at the molecular and cellular levels	Freeze-drying method	nZrO_2_ (<100 nm), nHAp (<200 nm)	These scaffolds helped in differentiation of osteoblast which were further enhanced with miR-590-5p presence on mouse mesenchymal stem cells	96
Gaihre B *et al.* 2018	Bone regeneration	Scaffolds of chitosan were hybridized with nano-calcium zirconate (CS-nCZ), ZrNp and nano-hydroxyapatite (CS-nHA)	To compare the physical and biological properties of these three scaffolds	Conventional freeze-drying technique	nZrO (22–50 nm), nCZ (7–25 nm), and nHA (30–90 nm)	The physical properties were comparable among the three scaffolds, but significantly higher compared to CS alone scaffolds. Zirconia based bioceramic materials had a lower response of osteoblasts. CS-nCZ had an increased rate of growth of pre-osteoblasts in comparison with CS-nZrO	129
Balagangadharan K *et al.* 2018	Bone regeneration	The composition of scaffolds was – (1) nano-hydroxyapatite (nHAp), (2) ZrNp and (3) chitosan (CS)	Evaluation of the additive impact of scaffolds of CS/nHAp/nZrO_2_ along with miR-590-5p on osteogenic potential at the molecular and cellular levels	Freeze–drying method	nZrO_2_ (<100 nm), nHAp (<200 nm)	These scaffolds helped in differentiation of osteoblast which were further enhanced with miR-590-5p presence on mouse mesenchymal stem cells	96

Introducing nanofillers, such as ZrNp into polymer matrix give rise to a new class of scaffold materials that are viable candidates to replace conventional autograft, allograft and xenografts. The various configuration with differing polymer materials and nanofillers did not allow for meaningful comparison, thus a research gap exists in the optimal combination of polymer blend that is conducive to bone regeneration.

### 4.5. Radiopacifying biomaterial

The radiopacity of dental materials is defined as an optical density value. It is usually expressed in eq. Al thickness (mm) for collating with other studies.^97^ The radiopacity of dental materials is crucial for differentiating dental restorative materials from teeth and adjacent structures.^98–100^ It also aids in the assessment of absorption of materials in bone structures, dissolutions of dental cements and its marginal adaptation. This is possible due to the varying radiopacity levels of these materials.^101,102^ Hence radiopacifiers are an integral component of innumerable dental materials; an example being calcium–silicate based materials. Calcium–silicate based materials have enjoyed immense popularity in the field of dentistry due to its versatility.^103^ Specifically, mineral trioxide aggregate (MTA) is widely used in various endodontic procedures such as pulp capping and pulpotomy, repair of perforation and root resorption defects, apexification and root end filling material during apicoectomy.^104^ The principal component of MTA is Portland cement, with bismuth oxide (Bi_2_O_3_) added as radiopacifiers.^105^ Bismuth oxide had been proven to adversely affect the performance of MTA. In the presence of Bi_2_O_3_, the hydration mechanism of MTA and precipitation of calcium hydroxide is impaired. Moreover, Bi_2_O_3_ particles serve as flaw that disrupt the cement matrix, leading to greater porosity and solubility of the final product.^106^ To circumvent these shortcomings, research is underway to search for new radiopacifying agents. Zirconium oxide (ZrO_2_) is a promising candidate that had produced satisfactory results in experimental studies.^107^ Therefore, this review intended to explore the characteristics of Portland cement (PC) modified with different fillers, with emphasis on ZrO_2_ and nanofillers. MTA's antibacterial efficacy comes from its high pH environment. It was proposed that addition of zinc oxide (ZnO) can enhance a material's antimicrobial activity.^108^ However, this claim was contradicted by a study that discovered that PC combined with ZnO nanoparticles did not result in significant reduction in *E. faecalis* count.^109^ In contrast, the ZnO nanoparticles decreased the compressive strength of PC. The same study also revealed that ZnO or ZrO_2_ can provide Portland cement with a radiopacity between 3.5–3.92 mm_Al_, which fulfilled the criteria set forth by ANSI/ADA.^109^ ZrO_2_ had the advantage of supplying radiopacity without the weaknesses associated with bismuth oxide. These include delayed setting time and retarded hydration.^110^ Niobium oxide was another material that was investigated for Portland cement modification. As with ZrO_2_, niobium oxide managed to impart Portland cement with a satisfactory radiopacity.^111^ MTA achieved the highest pH values and radiopacity, but from a clinical standpoint, the other materials attained functional radiopacity and comparable antibacterial efficacy as MTA.^111^ As the disadvantages of bismuth oxide in MTA became apparent, researchers are on the lookout for suitable replacements. Among these, ZnO, ZrO_2_ and Nb_2_O_5_ were the targets of multiple *in vitro* and *in vivo* studies. ZrO_2_ had emerged as the forerunner to replace bismuth oxide as it establishes acceptable radiopacity while preserving the cement's compressive strength, setting time and antimicrobial potency (Table 6).

**Table 6:** Application of ZrNp as radiopacifying biomaterial

Author/year	Application	Type of zirconia	Objective	Method of preparation	Particle size (nm)	Outcome	Ref.
Tanomaru *et al.* 2014	Restorative dentistry (endodontics)-Radiopacity	30% ZrO_2_ microparticles, 5–10% ZnO nanoparticles	To investigate the antibiofilm activity, compressive strength and radiopacity of Portland cement associated with ZrO_2_ or ZnO particles	—	—	Addition of ZrO_2_ microparticles and ZnO nanoparticles improved the radiopacity, decreased the compressive strength and maintain the antimicrobial activity of Portland cement	47
Li and Coleman, 2019	Restorative dentistry (endodontics)-Radiopacity	Micron-sized zirconium oxide (ZrO_2_) and bismuth oxide (Bi_2_O_3_) particles	To explore the early hydration chemistry and microstructure of white Portland cement blended with 20 wt% of ZrO_2_ and Bi_2_O_3_	Manual mixing of the cement with distilled water and metal oxide radiopacifiers at a fixed ratio	ZrO_2_ – 5 μm, Bi_2_O_3_ – 2 μm	ZrO_2_ improves the radiopacity of Portland cement-based materials without the disadvantages of increased setting time and retarded hydration that were found with bismuth oxide fillers	110
Tanomaru *et al.* 2014	Restorative dentistry (endodontics)-Radiopacity	Zirconium oxide and niobium oxide nanoparticles	To study the radiopacity, pH and antimicrobial properties of calcium silicate cements with added ZrO_2_ and Nb_2_O_5_	Polymeric precursor method	ZrO_2_ – 74 nm, Nb_2_O_5_ – 83 nm	ZrO_2_ and Nb_2_O_5_ promoted radiopacity, antimicrobial activity and alkalinity, supporting their use as radiopacifying agents for Portland cement	111

Hence, it can be construed that nano zirconia can serve as viable radiopacifying agents to be combined with calcium silicate cement in order to deliver an acceptable degree of radiopacity without the drawbacks of more commonly used bismuth oxides such as increased porosity and solubility.

## 5. Disadvantages of other biomaterials

Zirconia and nanotechnology are making headway in dental implantology. Bone graft materials such as hydroxyapatite and tricalcium phosphate, have been widely used owing to their excellent bioconductivity and biocompatibility.^112–116^ However, their use is limited as a bone graft material around implants owing to low strength and fracture toughness. It had been reported that a composite coating of zirconia and hydroxyapatite/titanium alloy exhibited a complex surface topography at the micro- and nano-structural level, which dramatically enhanced the osteoblastic cell activity and osseointegration.^117^ ZrNp is widely used in fabrication of fixed prosthesis such as crowns and bridges and holds promise as a denture base material. Traditionally polymethyl methacrylate is used, but it's lack of impact and flexural strength limits its service life in sudden drops and masticatory accidents. It has been demonstrated that ZrNp promote better strength and fracture toughness due to tetragonal to monoclinic phase transformation and microcrack nucleation.^61^

## 6. Toxicity of ZrNp

Research studies have shown that nano-zirconia is biocompatible when it is free of radioactive ingredients.^118^ They normally do not depict any adverse reactions as they are inert materials. Nanoparticles at higher concentrations and for more exposure time exhibit toxic effects. Similar reports have been seen with concentration dependent toxicity of ZrNp due to its oxidative stress caused by reactive oxygen species. Concentration of 100 μg ml^−1^ of ZrNp depicts inhibition of osteoinductive characteristics.^119^ Research have shown that ZrNp have detrimental effect on DNA of human dermal epithelial cells and apoptosis.^120^ Another study depicted apoptosis action impacting osteogenesis effect of osteoblast like cells due to high amount of oxidative damaging effect on the cells.^119^ Similar effect of ZrNp have been seen oxidative injury after intravenous injection in mice.^121^ Studies have shown that there could be possible toxic effects of ZrNp on the liver tissue after administration of a particular dose over a period of specific time on long-term basis. Another research has shown that rats when exposed to high dose of ZrNp, the concentration of liver enzyme elevated significantly. The acquired findings showed the substantial role of ZrNp as an reactive oxygen species agent and the ROS generates the formation of free radicals.^122^ ZrNp toxicity could be due to oxidative stress, cell apoptosis, lipid accumulation and activation of Akt-facilitated signaling pathway and changes in the gene expression.^123^ However, the scientific foundation for the toxic effects of ZrNp is inadequately explicated, and the perception of the various mechanisms is still limited, presenting far-reaching questions for their practical usage. Also, majority of long-term research done for testing cytotoxicity lacks the randomization and varies in the *in vitro* settings of the methodology. Overall ZrNp shows lesser toxic effects compared to titanium oxide and alumina.^124,125^

## 7. Conclusion

Zirconia is a versatile biomaterial that has important applications in various aspects of dentistry. By combining zirconia with nanotechnology, it is hoped that a superior class of bioactive materials can be produced. The performance of dental composite modified with ZrNp had been encouraging. Addition of ZrNp to composites and adhesives enhances their mechanical properties.

Use of ZrNp coated dental implant surfaces potentiates better osseointegration due to increased osteoblastic activity. Moreover, the composite coating of zirconia and titanium displayes good mechanical characteristics with a combined strength of 17 GPa.^126^ Radiopacity quality is of cements is improved by addition of ZrNp.

It has been demonstrated that ZrNp promotes better fracture toughness and prevents crack propagation of prosthetic materials^61^ and is evident by the high survival rate, lack of chipping/fracturing of veneering ceramic and good biologic reaction of fixed dental prosthesis.^62^

It can be concluded that ZrNp has produced very promising results in many *in vitro* studies across different areas of dentistry, but more longitudinal clinical studies are required before this technology can be unequivocally adopted in evidence-based practice.

## 8. Future perspective

As per critical analysis presented in this review, ZrNp due to its superior properties, can be used as it can be utilized as restorative, prosthetic, implant, bone regenerative and radiopacifying biomaterial for various dental applications. Tissue engineering is the tenet of regenerative dentistry. Zirconia has since emerged as a candidate material in the fabrication of bio-composite scaffold, which could potentially replace more conventional means of bone regeneration such as autografts and allografts. Relatively sparse number of investigations about the interaction between ZrNp and stem cells provides us the need to explore this area. Zirconia could be used as an endodontic post material with an ever-increasing demand for translucent all ceramic restorations. Additionally, zirconia does not produce corrosive products which cause metallic taste and oral sensitization. Usage zirconia brackets in orthodontics, which have high toughness and good sliding properties on both stainless steel and nickel–titanium arch wires along with less plaque retention could be explored further. ZrNp coated mini implants can be used in alignment of teeth due to its better stability and fixation within the bone. Zirconia coated instruments can be developed for periodontal and peri-implant treatment procedures for elimination of infections. Thus, still there exists a tremendous scope of exploring applications of ZrNp in dental applications.

## List of abbreviations

ZrO_2_: Zirconium oxide
Y-TZP: Tetragonal zirconia polycrystal
Y_2_O_3_: Yttria
ZrNp: Nano zirconia
LTD: Low temperature degradation
GTE: 3-Component monomer solution
*E. coli*
: *Escherichia coli*
*E. fecalis*
: *Enterococcus faecalis*
IZ: In-ceram zirconia
DZ: DC-zirkon
Y_2_O_3_ TZP: YZ
FPD: Fixed partial dentures
MPC: Magnetic pulsed compaction
CTE: Coefficient of thermal expansion
Ce-TZP/Al_2_O_3_: Zirconia/alumina composite
HF: Hydrofluoric acid
SEM: Scanning electron microscopy
Ti: Titanium
Zr: Zirconia
XPS: X-ray photoelectron spectroscopy
HAp: Hydroxyapatite
nZrO_2_: Nano-zirconium dioxide
nHAp: Nano-hydroxyapatite
CS: Chitosan
MTA: Mineral trioxide aggregate
PC: Portland cement
Bi_2_O_3_: Bismuth oxide
ZnO: Zinc oxide
Nb_2_O_5_: Niobium oxide

## Conflicts of interest

There is no conflict of interest and disclosures associated with the manuscript.

## Supplementary Material
